# Phosphorylation of the cell wall hydrolase MltG in response to cell wall stress modulates resistance toward cephalosporins in *Enterococcus faecalis*

**DOI:** 10.1128/jb.00099-25

**Published:** 2025-07-14

**Authors:** Alexis A. U. Knotek, Christopher J. Kristich

**Affiliations:** 1 Department of Microbiology and Immunology, Center for Infectious Disease Research, Medical College of Wisconsin 735651, Milwaukee, Wisconsin, USA; University of Illinois Chicago, Chicago, Illinois, USA

**Keywords:** MltG, protein phosphorylation, PASTA kinase, signal transduction, *Enterococcus*

## Abstract

*Enterococcus faecalis* is an opportunistic pathogen that colonizes the human gut microbiome. *E. faecalis* commonly establishes infection subsequent to antibiotic therapy in patients due to intrinsic resistance exhibited by *E. faecalis* toward cephalosporins and acquired resistance to many clinically used antibiotics. Intrinsic resistance toward cephalosporins in *E. faecalis* depends on the kinase activity of IreK, a transmembrane serine/threonine PASTA kinase that mediates responses to cell wall stress, including stress caused by cephalosporins, by phosphorylating downstream effector proteins. Our previous phosphoproteomics analysis identified MltG, a transmembrane protein with an extracellular catalytic domain that cleaves nascent peptidoglycan strands, as a putative substrate for phosphorylation by IreK in *E. faecalis*, suggesting the hypothesis that IreK-mediated phosphorylation of MltG might regulate cell wall homeostasis and possibly intrinsic cephalosporin resistance. Here we report that MltG is a bona fide direct substrate of IreK in *E. faecalis*. We found that MltG phosphorylation *in vivo* is enhanced in response to cell wall stress in an IreK-dependent manner, requiring a specific residue in the MltG cytoplasmic domain for phosphorylation by IreK both *in vivo* and *in vitro*. Finally, phosphoablative and phosphomimetic substitutions of MltG reciprocally influence resistance of *E. faecalis* to ceftriaxone, pointing to functional consequences of MltG phosphorylation. Collectively, our results reveal a novel pathway by which IreK senses antibiotic-mediated cell wall stress and responds by phosphorylating the cytoplasmic segment of MltG to enhance antibiotic resistance.

**IMPORTANCE:**

Infections caused by *Enterococcus faecalis* are increasingly prevalent and difficult to treat due to the multi-drug resistance exhibited toward common antibiotics. A greater understanding of the mechanisms underlying antibiotic resistance can enable the development of new drugs or strategies to overcome antibiotic-resistant infections. *E. faecalis* exhibits intrinsic resistance toward cephalosporins. This intrinsic resistance requires activity of the PASTA kinase IreK; however, few substrates for phosphorylation by IreK have been rigorously identified. Here, we report that MltG is directly phosphorylated by IreK in response to cell wall stress. This phosphorylation event acts to promote cephalosporin resistance as part of the IreK signaling network. Our results thereby validate a new substrate and expand knowledge of the IreK signaling pathway contributing to cephalosporin resistance.

## INTRODUCTION

*Enterococcus faecalis* is a species of gram-positive bacteria that is a commensal of the healthy gut microbiome. However, *E. faecalis* is also an opportunistic pathogen which can transition from its commensal state to cause serious infections (1). These infections are often difficult to treat due in part to intrinsic and acquired resistance of *E. faecalis* toward many clinically used antibiotics (2, 3). *E. faecalis* exhibits intrinsic resistance toward cephalosporins, a class of β-lactam antibiotics (4). Prior treatment with cephalosporins, which depletes the gut of susceptible microbes, is a known risk factor for the establishment of enterococcal infections (5, 6) because intrinsically cephalosporin-resistant *E. faecalis* can proliferate within the gastrointestinal tract and disseminate to other sites to establish infection (7–10). Therefore, further understanding of the mechanisms of intrinsic cephalosporin resistance of *E. faecalis* is needed to enable the prevention and treatment of enterococcal infections.

Intrinsic cephalosporin resistance of *E. faecalis* requires activation of the cell wall stress-responsive kinase, IreK (11–13). Detection of cell wall stress by IreK results in autophosphorylation and activation of its intracellular kinase domain, enhancing the ability of IreK to phosphorylate downstream substrates (12, 14). Previously, two IreK substrates, IreB and CroS, had been shown to contribute to enterococcal cephalosporin resistance in response to phosphorylation by IreK (15, 16). A phosphoproteomics study (17) identified additional putative substrates for phosphorylation by IreK, including GpsB, which has since been shown to be an authentic IreK substrate and to regulate cephalosporin resistance in a phosphorylation-dependent manner (13, 17, 18). The same phosphoproteomics study identified four residues in the cytoplasmic domain of the peptidoglycan transglycosylase MltG (Fig. S1) as potential sites of IreK-dependent phosphorylation (17).

We previously found that MltG of *E. faecalis* cleaves nascent peptidoglycan, as observed with MltG homologs (19–23), and either deletion of *mltG* entirely or impairment of MltG catalytic activity results in hyperresistance toward ceftriaxone, a representative cephalosporin (24). Most MltG homologs are integral membrane proteins: they contain a transmembrane domain near their N-terminus and two extracellular domains, the membrane-proximal LysM putative peptidoglycan-binding domain and the C-terminal YceG domain that catalyzes cleavage of nascent peptidoglycan (Fig. S1). A few MltG homologs, including MltG from *E. faecalis*, also contain an extended N-terminal cytoplasmic segment. The cytoplasmic domain of *E. faecalis* MltG is predicted by MobiDB-lite to be intrinsically disordered and has no known function, but contains the putative IreK-dependent phosphorylation residues. One residue in the cytoplasmic domain of MltG from *Streptococcus pneumoniae* has been identified as a putative substrate of the pneumococcal IreK homolog, StkP; however, the functional consequences of MltG phosphorylation have yet to be described for any MltG homolog (25).

In this study, we validated that MltG is a bona fide direct substrate of IreK in *E. faecalis*. We found that MltG phosphorylation *in vivo* is enhanced in an IreK-dependent manner in response to cell wall stress imposed by multiple antibiotics. Moreover, although our prior phosphoproteomics data suggested four potential sites of phosphorylation on MltG, we found that MltG T20 was sufficient to account for all phosphorylation we observed both *in vivo* and *in vitro*. Finally, phosphoablative and phosphomimetic substitutions at MltG T20 reciprocally influence resistance of *E. faecalis* to ceftriaxone, pointing to functional consequences of MltG phosphorylation. Together, our results reveal a novel pathway by which IreK senses antibiotic-mediated cell wall stress and responds by phosphorylating the cytoplasmic segment of MltG to enhance cephalosporin resistance.

## RESULTS

### MltG phosphorylation is enhanced in response to stress in an IreK-dependent manner *in vivo*

**Fig 1:**
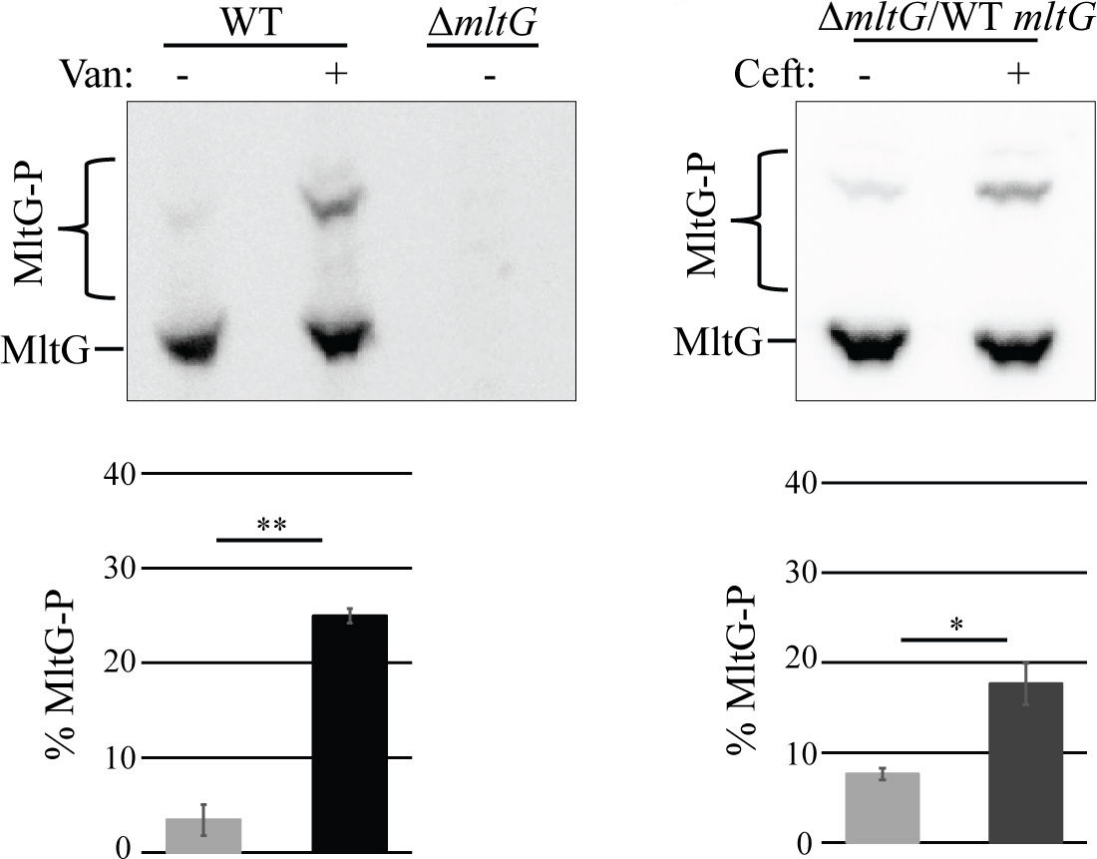
Antibiotic-induced cell wall stress induces MltG phosphorylation. Exponentially growing *E. faecalis* strains were exposed to 3 µg/mL vancomycin (van) or 128 µg/mL ceftriaxone (ceft) for 20 min, and whole-cell lysates were prepared. Lysates were subjected to phos-tag SDS-PAGE with immunoblotting for MltG. The upper bands represent phosphorylated proteoforms. Signal intensities from the phosphorylated and unphosphorylated bands were used to calculate % phosphorylation of MltG, presented as the average % phosphorylation in bar graphs. *n* = 3 and error bars represent ±s.d. *P < 0.05, **P < 0.01. ns, not significant. Student’s *t*-test (heteroscedastic, two tailed). Strains were wild type (WT) = OG1; Δ*mltG* = JL650; Δ*mltG/*WT *mltG* = JL650(pAAU12).

We previously demonstrated that MltG can be phosphorylated in an IreK-dependent manner in growing cells of *E. faecalis*.(17) Because IreK enhances phosphorylation of its substrates in response to various forms of cell wall stress (14), we hypothesized that MltG phosphorylation would increase upon treatment with cell wall-active antibiotics. We assessed MltG phosphorylation using phos-tag SDS-PAGE as previously described (13, 17), which allows for the separation of phosphorylated and non-phosphorylated protein proteoforms, where the unphosphorylated proteoform migrates most rapidly through the gel, and phosphorylated proteoforms migrate more slowly. We found that MltG phosphorylation was enhanced upon cell wall stress induced by exposure of *E. faecalis* to either vancomycin or ceftriaxone (Fig. 1), as reflected in the increased abundance of the more slowly migrating phosphorylated MltG proteoforms. Although the pattern of phosphorylated MltG proteoforms observed was essentially indistinguishable for cells treated with either vancomycin or ceftriaxone, for unknown reasons, vancomycin treatment resulted in a more robust MltG phosphorylation response. We therefore chose to use vancomycin treatment as our method of inducing cell wall stress for subsequent experiments.

**Fig 2:**
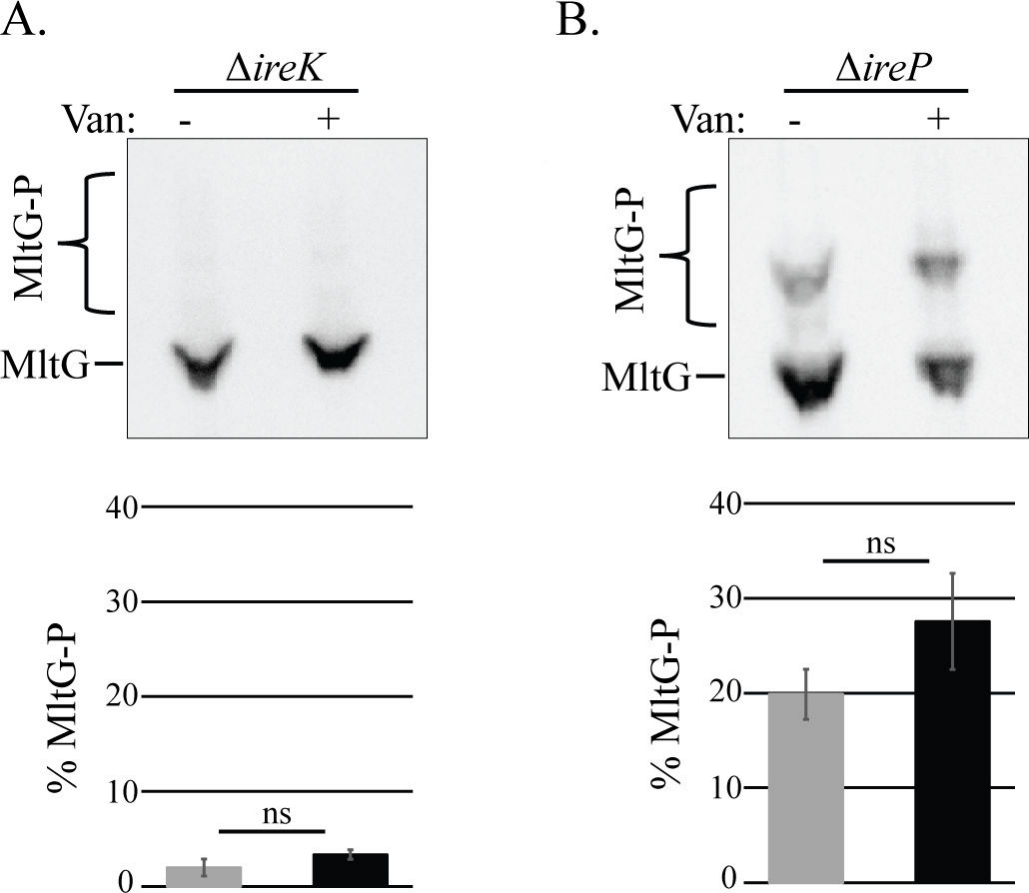
IreK is required for enhanced MltG phosphorylation in response to cell wall stress. Exponentially growing *E. faecalis* (**A**) Δ*ireK* and (**B**) Δ*ireP* cells were exposed to 3 µg/mL vancomycin (van) for 20 min, and whole-cell lysates were prepared. Lysates were subjected to phos-tag SDS-PAGE with immunoblotting for MltG. The upper bands represent phosphorylated proteoforms. Signal intensities from the phosphorylated and unphosphorylated bands were used to calculate percent phosphorylation of MltG, presented as the average percent phosphorylation in bar graphs. *n* = 3 and error bars represent ±s.d. ns, not significant. Student’s *t*-test (heteroscedastic, two tailed). Strains were (**A**) Δ*ireK =* JL206 and (**B**) Δ*ireP* = JL455.

To test if enhanced MltG phosphorylation observed in response to stress depends on IreK, we examined MltG phosphorylation in the Δ*ireK* and Δ*ireP* mutants in the presence of stress. IreP is the cognate phosphatase for IreK, and deletion of IreP results in IreK hyperactivity (11, 14), even in the absence of exogenous antibiotic-mediated stress. We found that in the Δ*ireK* strain, MltG was not phosphorylated even in response to stress (Fig. 2A). Conversely, in the Δ*ireP* mutant, MltG was already substantially phosphorylated in the absence of stress, and phosphorylation did not significantly increase in the presence of stress (Fig. 2B). Similar findings were observed in an evolutionarily distinct strain of *E. faecalis*, the vancomycin-resistant clinical isolate V583 (Fig. S2A), indicating that IreK-mediated enhancement of MltG phosphorylation in response to cell wall stress is broadly conserved across *E. faecalis*. We noted that the phosphorylated proteoform of V583 MltG migrated more rapidly during phos-tag SDS-PAGE than the phosphorylated proteoform of OG1 MltG. Although the underlying cause for this difference in mobility remains unknown, this phenomenon appears to be determined by the identity of the residue at position 24 in MltG itself, which is K24 in OG1 and N24 in V583 (the only difference in the protein sequences between OG1 and V583), because expression of the OG1 and V583 MltG variants in the reciprocal host resulted in the mobility of the phosphorylated proteoform that matched the mobility observed in the original host. For example, expression of the V583 MltG variant (whose phosphorylated proteoform migrates more rapidly in V583) in the OG1 host also resulted in a more rapidly migrating phosphorylated proteoform (Fig. S2B).

### MltG phosphorylation requires T20 *in vivo*

**Fig 3:**
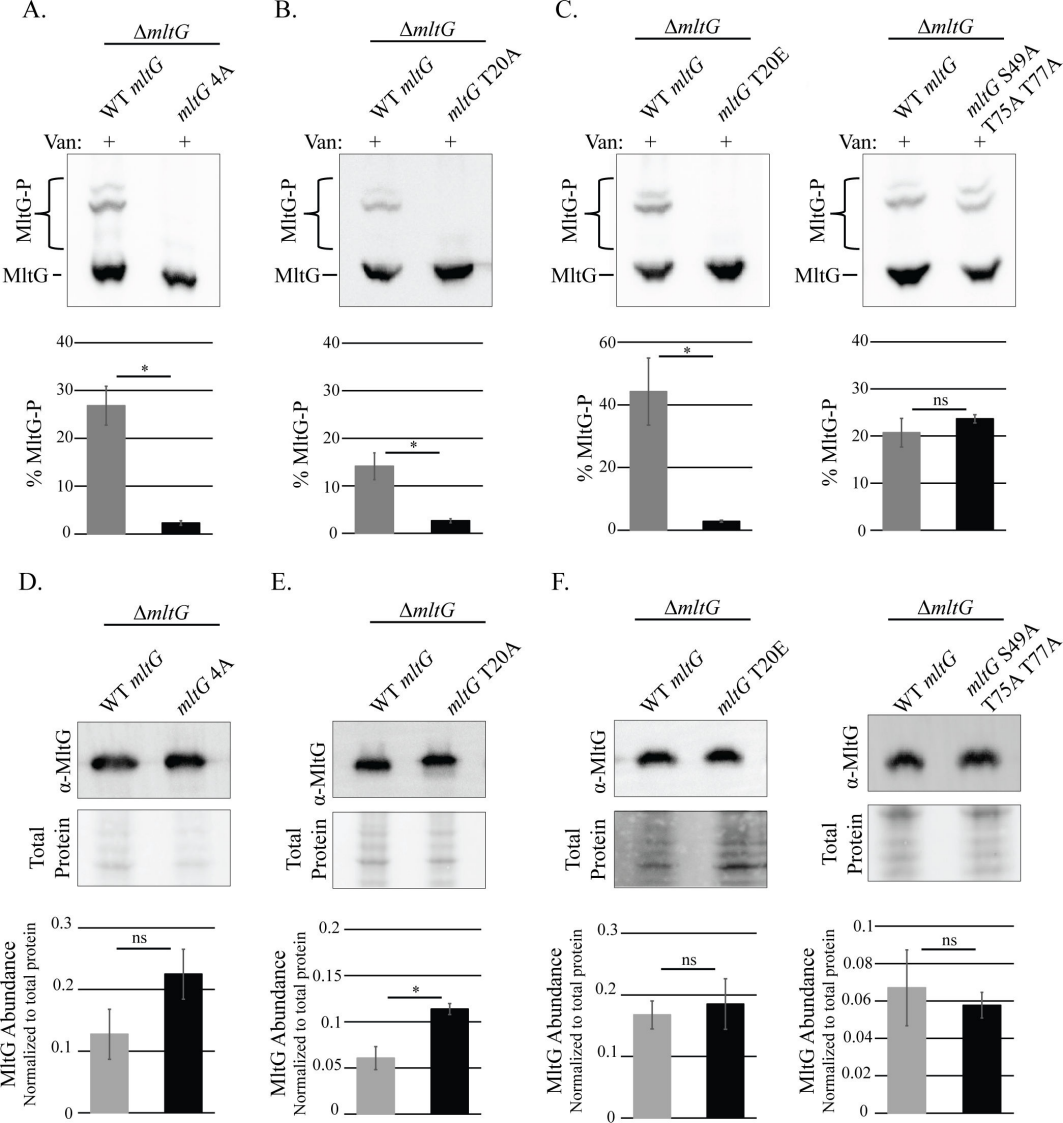
MltG T20 is required for antibiotic-induced MltG phosphorylation *in vivo*. (**A–C**) Phosphorylation of ectopically expressed MltG variants was compared to phosphorylation of WT MltG expressed from the same vector by phos-tag SDS-PAGE. Exponentially growing *E. faecalis* strains were exposed to 3 µg/mL vancomycin (van) for 20 min, and whole-cell lysates were prepared. Lysates were subjected to phos-tag SDS-PAGE with immunoblotting for MltG. The upper bands represent phosphorylated proteoforms. Signal intensities from the phosphorylated and unphosphorylated bands were used to calculate percent phosphorylation of MltG, presented as the average percent phosphorylation in bar graphs compared to WT *mltG* expressed from the same plasmid. (**D–F**) Ectopic expression of MltG from plasmids was confirmed by subjecting whole-cell lysates to SDS-PAGE supplemented with 2,2,2-trichloroethanol for total protein detection and immunoblotting with MltG antiserum. Expression of all MltG mutants was compared to that of WT *mltG* expressed from the same plasmid. *n* = 3 and error bars represent ±s.d. *P < 0.05. ns, not significant. Student’s *t*-test (heteroscedastic, two tailed). Strains were Δ*mltG/*WT *mltG* = JL650(pAAU12), (**A and D**) Δ*mltG*/*mltG* 4A = JL650(pAAK20), (**B and E**) Δ*mltG/mltG* T20A *=* JL650(pAAK16), and (**C and F**) Δ*mltG/mltG* T20E *=* JL650(pAAK45) and Δ*mltG/mltG* S49A T75A T77A *=* JL650(pAAK53).

Our phosphoproteomics study identified four putative sites of phosphorylation (T20, S49, T75, and T77) in the cytoplasmic domain of MltG (17). To determine if IreK-dependent phosphorylation of MltG requires one or more of these sites, a phosphoablative MltG 4A mutant (T20A S49A T75A T77A) was expressed from a plasmid in the Δ*mltG* mutant. As a control, we demonstrated that wild-type (WT) MltG was expressed and phosphorylated to the same extent as chromosomally encoded MltG when ectopically expressed from the plasmid in the Δ*mltG* mutant (Fig. S3A and B). The MltG 4A mutant was expressed at a similar level as wild-type MltG (Fig. 3D). However, phosphorylation of the MltG 4A mutant was almost completely absent, with the exception of a faint phosphorylation band that exhibited intermediate mobility between the two most prominent phosphorylation bands and unphosphorylated MltG (Fig. 3A). Therefore, phosphorylation of wild-type MltG is largely dependent on at least one of the potential phosphorylation sites identified by phosphoproteomics.

To determine if MltG phosphorylation required any specific site(s), a set of single alanine mutants in which individual phosphosites were substituted with alanine was constructed, expressed in the Δ*mltG* strain, and analyzed by phos-tag SDS-PAGE. We found that MltG S49A, T75A, and T77A mutants were expressed at levels similar to wild-type MltG (Fig. S4B) and did not significantly impact the pattern of phosphobands or the percentage of MltG phosphorylation (Fig. S4A). While MltG T20A was, for unknown reasons, present at a modestly higher abundance than wild-type MltG (Fig. 3E), this single phosphoablative mutant prevented phosphorylation to the same extent as the MltG 4A mutant (Fig. 3B), demonstrating that MltG phosphorylation is dependent on T20. This was also true for cells treated with ceftriaxone (Fig. S5), where the 4A and T20A MltG variants did not become phosphorylated, indicating that both vancomycin- and ceftriaxone-induced IreK activation lead to phosphorylation of MltG at T20.

Because our phosphoproteomics data suggested MltG could be phosphorylated at multiple sites, and because the T20A substitution appeared to eliminate most MltG phosphorylation, we hypothesized that T20 might serve as a “licensing” site for MltG phosphorylation, meaning that phosphorylation of MltG must first occur at T20 to enable phosphorylation of additional phosphosites, as previously observed for residue T133 of GpsB (18). To test this, we analyzed phosphorylation of a phosphomimetic T20E mutant which was expressed at levels similar to wild-type MltG (Fig. 3F). Mutation of phosphosites to glutamate can mimic the addition of a phosphoryl group at sites of phosphorylation (14, 18). Here, we saw that T20E did not result in any additional phosphorylation of MltG (Fig. 3C), suggesting that phosphorylation of T20 is not required to enable phosphorylation at additional sites. To test if MltG was only phosphorylated at T20 under the conditions tested, we constructed a 3A mutant (MltG S49A T75A T77A) which was expressed at a similar level as wild-type MltG (Fig. 3F), in which T20 is the only remaining site available for phosphorylation. The MltG 3A mutant was phosphorylated to the same extent as WT MltG (Fig. 3C), indicating that under these conditions, T20 is sufficient to support wild-type phosphorylation of MltG.

### MltG associates with IreK *in vivo*

**Fig 4:**
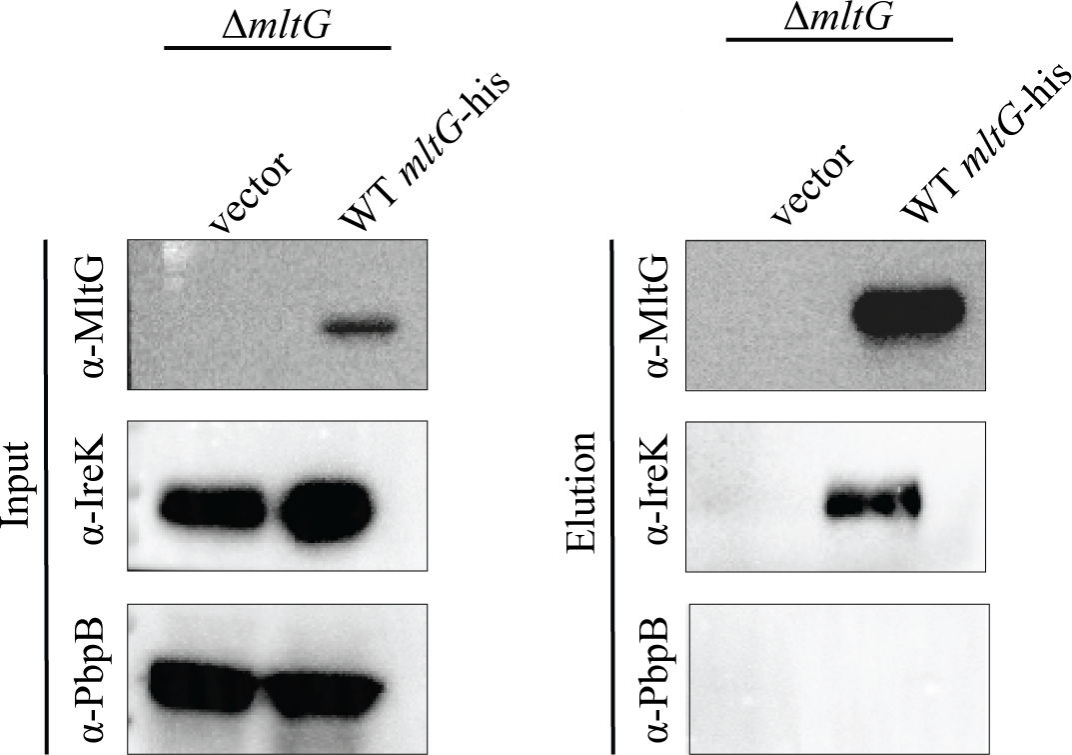
MltG associates with IreK *in vivo*. MltG-his was enriched from lysates of exponentially grown *E. faecalis* using immobilized metal affinity chromatography. Input and elution fractions were subjected to SDS-PAGE with immunoblotting for MltG, IreK, and PbpB. Data are representative of at least two independent experiments. Strains were Δ*mltG*/vector = JL650(pJRG9) and Δ*mltG*/WT *mltG-his* = JL650(pPS5).

To test if IreK-dependent phosphorylation of MltG *in vivo* could be due to direct phosphorylation of MltG by IreK, we probed for association of IreK with his-tagged MltG by co-purification from *E. faecalis* cell lysates. His-tagged MltG was demonstrated to be functional (Table S1) and was successfully enriched from cell lysates (Fig. 4). We found that IreK was present in the elution with MltG-his, while PbpB (another membrane protein to serve as a negative control) was not (Fig. 4). This suggests that IreK is in association with MltG and could directly phosphorylate MltG *in vivo*.

### MltG is directly phosphorylated by IreK *in vitro*

**Fig 5:**
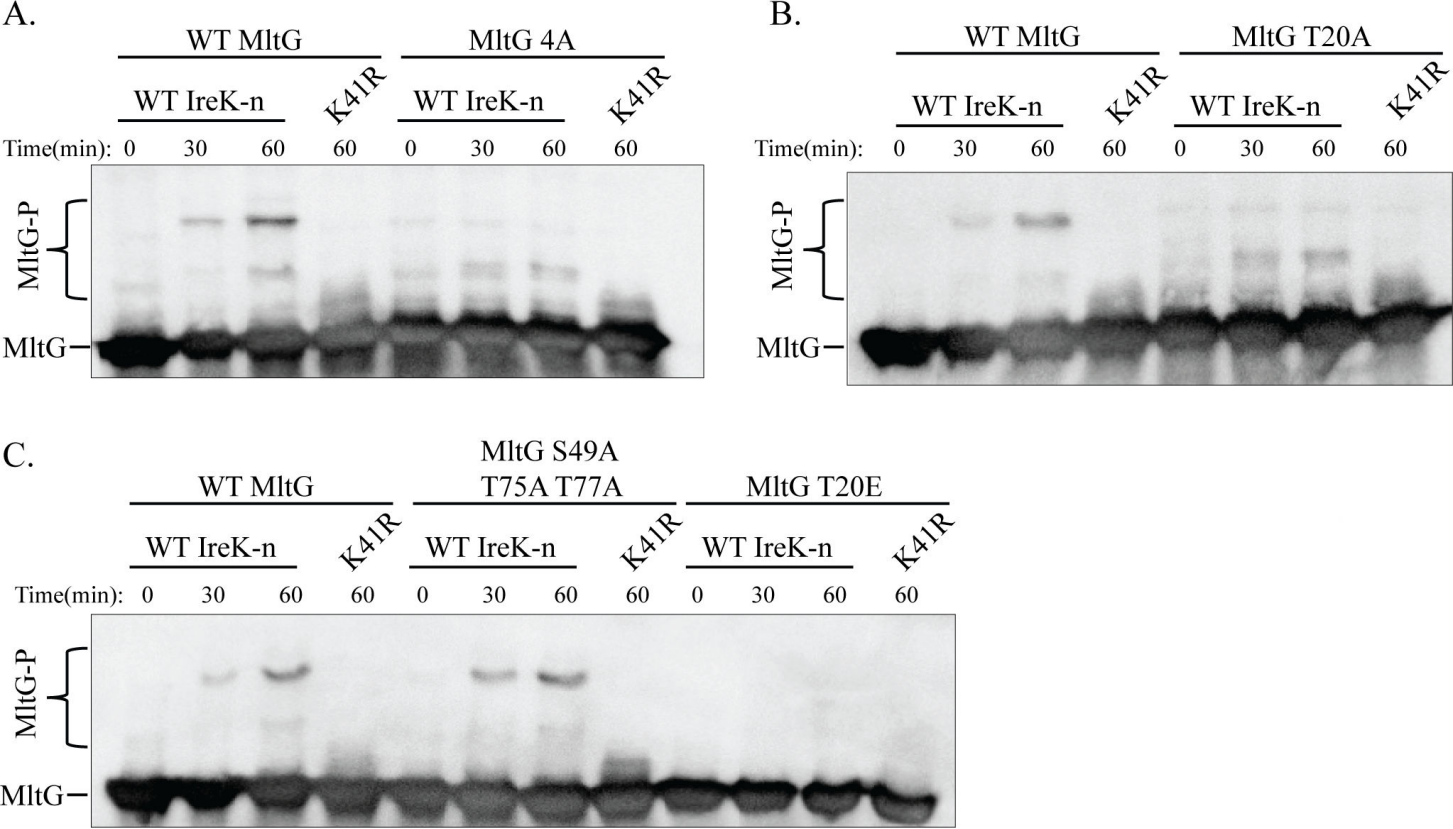
MltG is directly phosphorylated by IreK *in vitro*, dependent on MltG T20. Purified MltG variants were analyzed for phosphorylation by IreK using *in vitro* kinase reactions. WT MltG and (**A**) MltG 4A, (**B**) MltG T20A, and (**C**) MltG S49A T75A T77A and MltG T20E were combined with IreK-n (WT or K41R). After collecting the “time 0” sample, 2 mM ATP was added to initiate the reaction. At intervals, samples were quenched by addition of Laemmli buffer, boiled, and subjected to phos-tag SDS-PAGE with immunoblotting for MltG. Data are representative of three independent experiments.

To test if IreK phosphorylates MltG directly, we conducted *in vitro* phosphorylation assays using purified IreK-n (the isolated N-terminal kinase domain of IreK) as previously described (13, 15, 18), along with purified full-length MltG as the substrate. Reactions were subjected to phos-tag SDS-PAGE to assess MltG phosphorylation. At “time 0” prior to initiation of the kinase reaction, MltG exhibited little to no phosphorylation, indicating that MltG was purified from the *Escherichia coli* expression host in a non-phosphorylated form, as expected. MltG phosphorylation increased over time in the presence of wild-type IreK-n (Fig. 5A), revealed by the increasing signal from a prominent phosphorylated band. As a control, we demonstrated that a catalytically impaired mutant of IreK-n, K41R, did not phosphorylate MltG (Fig. 5A), confirming that no contaminants in the IreK-n or MltG preparations were responsible for MltG phosphorylation. These findings demonstrate that IreK directly phosphorylates MltG.

To determine if MltG T20 was required for phosphorylation of MltG *in vitro*, we analyzed MltG 4A and T20A variants using the same approach and found that these phosphoablative variants were virtually identical to each other and exhibited a loss of the most prominent IreK-induced phosphorylation band observed with wild-type MltG (Fig. 5A and B), although a less prominent phospho-MltG band exhibiting intermediate mobility between the most prominent IreK-induced band and unphosphorylated MltG was still present. As we had done with the *in vivo* studies above, we also tested whether T20 was sufficient for wild-type MltG phosphorylation by analyzing the MltG 3A mutant or if phosphorylation at T20 promoted additional phosphorylation by analyzing the MltG T20E mutant. We found that MltG 3A was sufficient for wild-type phosphorylation and that MltG T20E did not promote further phosphorylation of MltG (Fig. 5C). We also note that the most prominent IreK-induced phosphorylation band was absent when using MltG T20E, mimicking the findings with T20A, as would be expected because the T20E variant cannot be phosphorylated at that site. Together, these results are consistent with the *in vivo* results above and indicate that MltG T20 is required for phosphorylation of MltG by IreK.

### Impact of MltG phosphorylation on cephalosporin resistance

Given that MltG phosphorylation is enhanced in response to ceftriaxone-mediated stress (Fig. 1) and MltG impacts ceftriaxone resistance (Knotek and Kristich, in press), we tested how MltG phosphorylation impacted cephalosporin resistance by determining minimal inhibitory concentrations toward ceftriaxone for MltG T20A and T20E variants. Modest but reciprocal effects on ceftriaxone resistance were observed: the phosphoablative T20A mutant exhibited a decrease in ceftriaxone resistance, whereas the phosphomimetic T20E mutant exhibited an increase (Table 1). Together, these results suggest that IreK-mediated phosphorylation at T20 serves to promote resistance toward ceftriaxone, consistent with prior observations that IreK mediates adaptive responses to cell wall stress, such as exposure to ceftriaxone.

**TABLE 1:** Influence of MltG T20 substitutions on cephalosporin resistance

Strain^*a*^	Ceftriaxone MIC (µg/mL)^*b*^	
WT/vector	64	
ΔmltG/vector	512	
ΔmltG/ WT mltG	64	
	T→ A	T→ E
ΔmltG/mltG T20	32	128

^*a*^ Strains were WT/vector = OG1(pJRG9), Δ*mltG*/vector = JL650(pJRG9), Δ*mltG/*WT *mltG* = JL650(pAAU12), Δ*mltG/mltG* T20A *=* JL650(pAAK16), and Δ*mltG/mltG* T20E *=* JL650(pAAK45).

^*b*^ The median minimum inhibitory concentration (MIC) of ceftriaxone determined from at least three biological replicates.

### Impact of MltG phosphorylation on cell wall homeostasis

**Fig 6:**
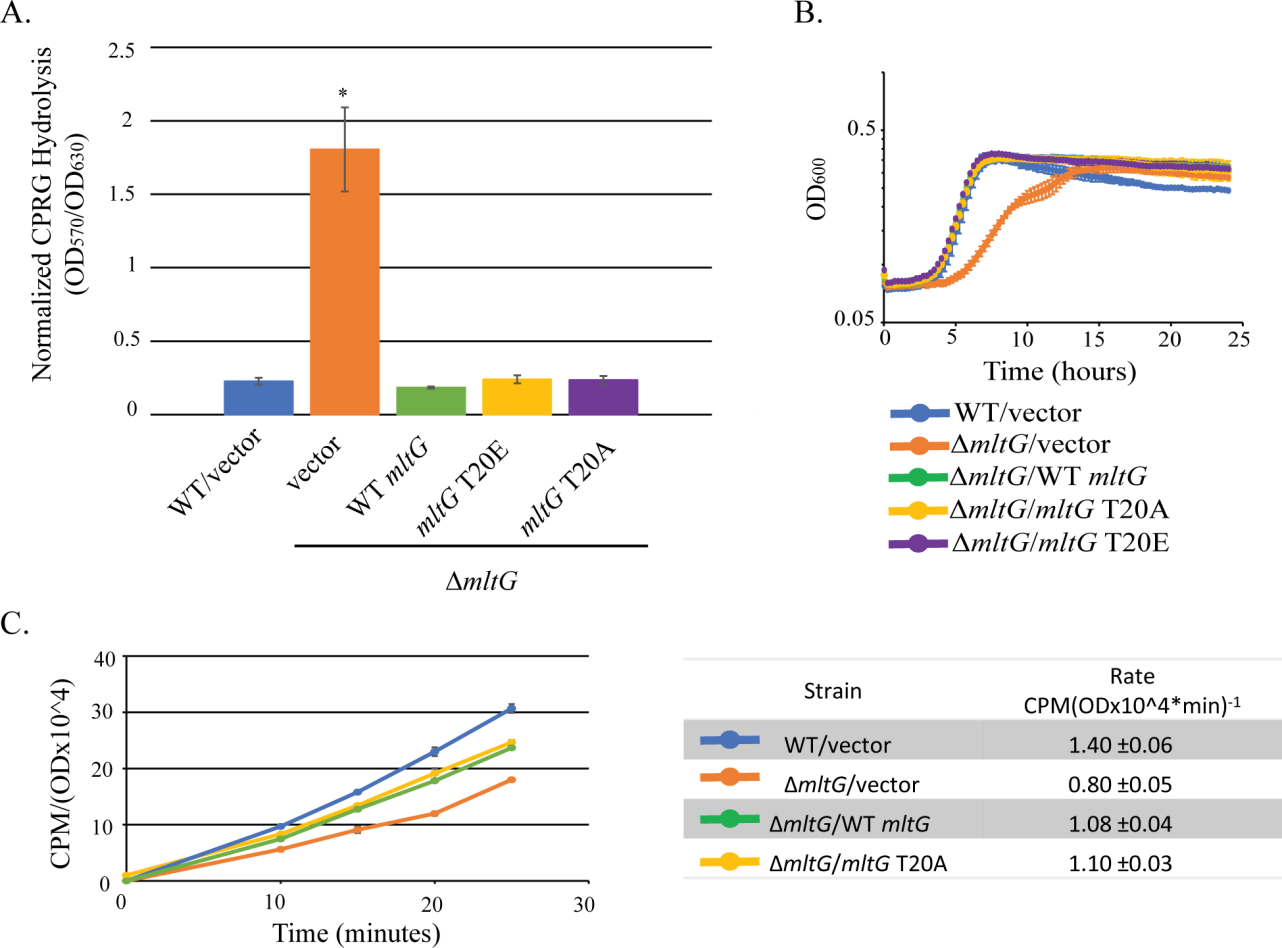
MltG T20 phosphomutants do not impair cell wall homeostasis. (**A**) β-galactosidase producing strains were grown for 24 hours in the presence of chlorophenol red-β-d-galactopyranoside (CPRG). The graph depicts cleavage of CPRG (OD_570_) normalized to bacterial growth (OD_630_). *n* = 3 and error bars represent ±s.d. *P < 0.05. Student’s *t*-test (heteroscedastic, two tailed) relative to WT/vector. (**B**) Bacterial growth (OD_600_) was monitored over the course of 24 hours. Data represent the mean ± s.d. of three biological replicates (two replicates for WT/vector). (**C**) The rate of PG synthesis during the exponential phase was determined by monitoring incorporation of ^14^C GlcNAc into peptidoglycan and normalizing to cell density. Label incorporation rates presented in the table were calculated from data points between 10 and 25 min. Data represent ±s.d. for two biological replicates. Strains were WT/vector = OG1(pJRG9), Δ*mltG*/vector = JL650(pJRG9), Δ*mltG*/WT *mltG* = JL650(pAAU12), Δ*mltG*/*mltG* T20A = JL650(pAAK16), and Δ*mltG*/*mltG* T20E = JL650(pAAK45).

To begin understanding how phosphorylation of MltG at T20 might impact cephalosporin resistance, we analyzed MltG T20 phosphoablative or phosphomimetic variants for several phenotypes that are often perturbed in *E. faecalis* mutants exhibiting altered resistance toward cephalosporins (including a mutant lacking MltG entirely): growth kinetics, cell wall integrity, and peptidoglycan synthesis (26–30). As previously reported (Knotek and Kristich, in press), deletion of *mltG* results in defects for each of these cellular phenotypes. However, we found that the T20 variants behaved essentially identical to wild-type MltG when analyzed for all of these traits: the T20 variants restored wild-type growth kinetics, cell wall integrity, and peptidoglycan synthesis to cells lacking wild-type MltG (Fig. 6A through C). Thus, it remains unclear how phosphorylation at T20 impacts cephalosporin resistance of *E. faecalis*.

## DISCUSSION

MltG homologs have been studied in a variety of bacterial species. Most MltG homologs are integral membrane proteins, containing a transmembrane domain near their N-terminus and two extracellular domains, the membrane-proximal LysM putative peptidoglycan-binding domain and the C-terminal YceG domain that catalyzes cleavage of nascent peptidoglycan. A few MltG homologs, including MltG from *E. faecalis*, also contain an extended N-terminal cytoplasmic segment of unknown function. Using phosphoproteomics, we previously identified four putative sites of IreK-dependent phosphorylation on the cytoplasmic segment of MltG in *E. faecalis* (17). Similarly, a phosphoproteomics study also identified MltG as a putative substrate of the IreK homolog, StkP, in *Streptococcus pneumoniae* (25). Only one putative phosphorylation site on pneumococcal MltG was identified (Thr155, not conserved in *E. faecalis*), and phosphorylation at that site has not been validated via orthogonal methods or functionally characterized by downstream approaches (25). Hence, phosphorylation of MltG may be more widespread than currently appreciated but is not known to be functionally important for any MltG homolog. Here, we found that *E. faecalis* MltG, (i) phosphorylation *in vivo* is enhanced in an IreK-dependent manner in response to cell wall stress imposed by multiple antibiotics with different mechanisms of action; (ii) phosphorylation occurs directly by IreK *in vitro*; and (iii) phosphorylation requires residue MltG T20 both *in vivo* and *in vitro*. IreK and MltG were found to associate within exponentially growing cells, consistent with the finding that MltG is a bona fide substrate of IreK in *E. faecalis* cells. Phosphoablative and phosphomimetic substitutions at MltG T20 reciprocally influence resistance of *E. faecalis* to ceftriaxone, pointing to functional consequences of MltG phosphorylation. Overall, these results are consistent with the model that IreK senses antibiotic-mediated cell wall stress and responds by phosphorylating substrates, including the cytoplasmic segment of MltG, to enhance antibiotic resistance.

Two discrepancies are apparent between our previous phosphoproteomics study and the results in this work. First, three potential phosphorylation sites on MltG (S49, T75, and T77) were detected by phosphoproteomics, but mutations at those sites (individually or together) do not affect MltG phosphorylation as detected by phos-tag SDS-PAGE in this study. One possibility to explain this discrepancy is that residues S49, T75, and T77 are only phosphorylated on a small fraction of MltG molecules, insufficient to produce a unique and detectable band after phos-tag SDS-PAGE and immunoblotting. Another possibility is that differences in experimental conditions might lead to differences in the MltG residues that are phosphorylated. For example, for the phosphoproteomics analysis, *E. faecalis* cells were cultured in MM9YE + glucose medium, whereas in the current study, cells were cultured in Mueller-Hinton broth (MHB) medium. It is currently unknown if the growth medium affects phosphorylation of substrates by IreK, but the possibility cannot be ruled out. A second discrepancy is suggested by the presence (albeit faint) of a slightly more slowly migrating, potentially phosphorylated band after phos-tag SDS-PAGE analysis of the MltG 4A mutant (Fig. 3). If this faint band represents a phosphorylated proteoform of MltG, that would mean a residue not detected by phosphoproteomics can be phosphorylated. This additional MltG phosphoproteoform is observed at relatively low abundance and may therefore be below the limit of detection within the phosphoproteomics study, which did not use vancomycin (an antibiotic used here due to robust MltG phosphorylation observed by phos-tag SDS-PAGE) to induce IreK substrate phosphorylation.

One of the four potential sites of MltG phosphorylation identified in our previous phosphoproteomics study was MltG T20. Our results indicate that, at least under the conditions of our experiments, T20 is the primary contributor to MltG phosphorylation in *E. faecalis* in response to both vancomycin and ceftriaxone exposure. An alanine substitution at T20 essentially completely eliminated MltG phosphorylation both *in vivo* and *in vitro*, whereas alanine substitutions at the other putative phosphorylation sites individually or in combination had no effect. To address the possibility that phosphorylation at T20 may “license” phosphorylation of other sites on MltG (as previously observed for another substrate of IreK, GpsB), we constructed a phosphomimetic mutant (MltG T20E) and analyzed its phosphorylation (18). We found that although the MltG T20E mutant was functional to promote cephalosporin resistance, MltG T20E did not result in additional phosphorylation of MltG. Additionally, a 3A mutant, in which all putative phosphosites are substituted with alanine except for T20, was able to support wild-type phosphorylation of MltG, together supporting a model in which MltG T20 is the only putative site at which phosphorylation occurs and is functionally important with regard to cephalosporin resistance under these conditions.

To begin understanding how phosphorylation at T20 influences ceftriaxone resistance, we analyzed growth kinetics, cell wall integrity, and peptidoglycan synthesis of *E. faecalis* strains expressing MltG with substitutions at T20, because these are phenotypes that are often altered in *E. faecalis* mutants exhibiting changes in cephalosporin resistance (including a mutant lacking MltG entirely). Specifically, we previously demonstrated that deletion of *mltG* is detrimental to cell wall homeostasis, as revealed by a reduction in cell wall integrity, peptidoglycan synthesis, and growth rate for the Δ*mltG* mutant. These defects ultimately drive cephalosporin resistance via activation of cell wall stress signaling systems (Knotek and Kristich, in press). However, expression of the MltG T20 variants did not affect any of these properties in a detectable manner (Fig. 6), suggesting that phosphorylation of MltG does not directly affect MltG function with regard to basic cell wall homeostasis. Hence, it remains unclear mechanistically how phosphorylation of MltG impacts cephalosporin resistance.

Based on previous studies, we speculate that phosphorylation might alter MltG localization and/or protein-protein interactions (PPIs) to influence MltG catalytic activity. We previously reported (Knotek and Kristich, in press) that expression of catalytically impaired MltG resulted in elevated ceftriaxone resistance of *E. faecalis* (but did not affect growth kinetics, cell wall integrity, or peptidoglycan synthesis). Moreover, both *Escherichia coli* and *Bacillus subtilis* MltG homologs exhibit increased catalytic activity *in vitro* when in the presence of active peptidoglycan synthases (21). Together, these observations suggest that phosphorylation at *E. faecalis* MltG T20 may negatively modulate MltG catalytic activity via altered MltG localization or PPIs to impact cephalosporin resistance. In such a model, improper localization of MltG or disrupted interaction with peptidoglycan synthases (due to T20E substitution) may result in partially impaired or inefficient MltG catalysis in cells, leading to elevated ceftriaxone resistance similar to that observed with the catalytically inactive MltG. As to how impaired MltG catalysis may result in elevated ceftriaxone resistance, we speculate that changes to the peptidoglycan composition might contribute. In *E. coli*, deletion of *mltG* results in increased length of peptidoglycan strands (19). Likewise, wild-type MltG was demonstrated to inhibit the crosslinking activity of peptidoglycan synthases *in vitro*, while a catalytically inactive MltG mutant did not exhibit this inhibitory effect (21). Thus, a potential increase in peptidoglycan strand length and/or crosslinks due to impaired MltG catalysis may fortify the cell wall, increasing resistance toward ceftriaxone. Future studies will explore these possibilities in an effort to understand how IreK-mediated phosphorylation of MltG in response to cell wall stress regulates antibiotic resistance in *E. faecalis*.

## MATERIALS AND METHODS

### Bacterial strains and growth conditions

Bacterial strains and plasmids used in this study are listed in Table S2. *E. coli* strains were grown using lysogeny broth or agar (Difco). *E. faecalis* spp. were grown using MHB or agar (Difco) at 30°C unless otherwise indicated. All cultures were grown aerobically with shaking (225 rpm). As appropriate, 10 µg/mL chloramphenicol or 50 µg/mL kanamycin was used for maintenance of plasmids.

### Construction of plasmids

All recombinant plasmids were constructed using Gibson assembly (31). The full insert of all constructs was sequenced to confirm the absence of mutations. MltG (wild type and mutants) was ectopically expressed in *E. faecalis* OG1 using the enterococcal expression plasmid pJRG9 under the control of the constitutive P23s promoter. For purification of recombinant MltG from *E. coli*, MltG (wild type and mutants) was expressed from the isopropyl β-D-1-thiogalactopyranoside-inducible expression vector pET28a-His-smt3, which encodes a His_6_-SUMO fusion.

### Whole-cell lysate preparation

Stationary-phase cultures were normalized to OD_600_ = 0.01 in MHB (supplemented with 10 µg/mL chloramphenicol when required for maintenance of plasmids) and grown to exponential phase, with shaking at 37°C. Cells were harvested by addition of an equal volume of ice-cold ethanol:acetone (1:1) and collected by centrifugation at 4,000 rpm at 4°C, followed by washing with 1 mL water and resuspension in lysozyme solution (10 mM Tris, 20% sucrose, 50 mM NaCl [pH 8.0]). Cells were normalized by OD_600_, treated with 5 mg/mL lysozyme for 20 min at 37°C, and mixed with 5× Laemmli buffer. Cell lysates were boiled for 5 min and then stored at −20°C until electrophoresis.

### Phos-tag SDS-PAGE

Phos-tag SDS-PAGE and immunoblot analysis of MltG was performed as previously described by Minton et al., except polyvinylidene fluoride (PVDF) membrane was used for MltG immunoblots and cell lysates were boiled as described above (13).

### MltG- IreK co-purification

Enrichment of MltG-his from *E. faecalis* cells was performed according to the method described by Nelson et al. (27), and the resulting samples were analyzed by immunoblotting.

### Purification of IreK-n

Recombinant wild-type His_6_-IreK-n kinase (intracellular domain only) and the catalytically impaired His_6_-IreK-n K41R mutant were purified from *E. coli* BL21(DE3) cells as described by Minton et al. (13).

### Purification of MltG

Recombinant His_6_-SUMO-MltG variants (wild type and phosphomutants) were expressed and purified from *E. coli* C43(DE3) cells solubilized in n-dodecyl-β-D-maltoside (DDM) as described previously (Knotek and Kristich, in press). The His_6_-SUMO tag was cleaved by Ulp1 protease, leaving only native MltG residues, prior to the use of MltG in phosphorylation reactions.

### *In vitro* phosphorylation assay

*In vitro* phosphorylation assays were performed as described by VanZeeland et al., with the following modifications (18). Prior to use in the reaction, purified recombinant MltG was dialyzed from its storage buffer (20 mM Tris, pH 7.5, 500 mM NaCl, and 0.01% DDM) into a reaction buffer (50 mM Tris, pH 7.5, 25 mM NaCl, and 0.01% DDM). MltG (3 µM WT or phosphomutant) was then incubated with 3.3 µM IreK-n (WT or K41R) in the reaction buffer supplemented with 5 mM MgCl_2_ for 5 min at 37°C. To initiate the phosphorylation reaction, 2 mM ATP was added to the reactions. At intervals, samples were taken from each reaction and quenched by the addition of 5× SDS-PAGE Laemmli buffer and boiled for 5 min. Samples were subjected to phos-tag SDS-PAGE and analyzed for MltG phosphorylation as previously described.

### Antimicrobial susceptibility assays and growth curves

Stationary-phase cultures were normalized to OD_600_ = 4 × 10^−5^ (~1 × 10^5^ CFU) and inoculated into 100-well honeycomb plates containing antibiotic for plasmid maintenance as appropriate and twofold serial dilutions of ceftriaxone. Cultures were grown for 24 hours at 37°C in a Bioscreen C plate reader. Growth was monitored as OD_600_, measured every 15 min with shaking before each measurement. The minimum inhibitory concentration was determined as the lowest concentration of ceftriaxone to inhibit growth over 24 hours. Growth curves were obtained from the samples prepared in the same manner, grown in the absence of ceftriaxone.

### Standard SDS-PAGE

Proteins were separated by SDS-PAGE supplemented with 2,2,2-trichloroethanol (TCE) at 150 V for 1 hour followed by activation of TCE using a ChemiDoc imaging system (BioRad) and transfer to PVDF membrane (BioRad) using a Transblot Turbo system (BioRad) at 25 V for 14 min. After transfer, TCE was imaged using a ChemiDoc imaging system, and membranes were blocked in 5% milk for 1 hour at room temperature. Membranes were probed with custom primary antiserum, diluted 1:5,000 in Tris-buffered saline with Tween 20, to detect MltG. Membranes were then probed with horseradish peroxidase-conjugated secondary antibody, diluted 1:5,000 in 5% milk, and imaged using a ChemiDoc imaging system.

### Cell wall integrity assessment

Cell wall integrity was assessed by chlorophenol red-β-d-galactopyranoside hydrolysis, as previously described (30).

### Rate of peptidoglycan synthesis

Peptidoglycan synthesis assays on exponentially growing *E. faecalis* cells were performed by monitoring the incorporation of ^14^C N-acetylglucosamine into peptidoglycan as previously described (26, 29, 32).

## SUPPLEMENTAL MATERIAL

Supplemental material jb.00099-25-s0001.pdf Fig. S1 to S4, Tables S1 and S2, and supplemental references.

ASM does not own the copyrights to Supplemental Material that may be linked to, or accessed through, an article. The authors have granted ASM a non-exclusive, world-wide license to publish the Supplemental Material files. Please contact the corresponding author directly for reuse.
