# Supplementary material for: Phosphorylation of the cell wall hydrolase MltG in response to cell wall stress modulates resistance toward cephalosporins in Enterococcus faecalis
Source: J Bacteriol. 2025 Jul 14;207(8):e00099-25. doi: 10.1128/jb.00099-25 (PMC12369325; doi:10.1128/jb.00099-25)
Supplement: Supplemental material — Fig. S1 to S4, Tables S1 and S2, and supplemental references. [file jb.00099-25-s0001.pdf]

Supplemental Material for:

Title: Phosphorylation of the cell wall hydrolase MltG in response to cell wall stress modulates resistance toward cephalosporins in *Enterococcus faecalis*

Authors: Alexis AU Knotek, Christopher J Kristich[#]

Running title: Phosphorylation of the cell wall hydrolase MltG

Affiliation:
Department of Microbiology and Immunology
Center for Infectious Disease Research
Medical College of Wisconsin
8701 Watertown Plank Rd
Milwaukee, WI 53226

#Address correspondence to Christopher J. Kristich, ckristich@mcw.edu.

```
MANDNQNNQDPKSSLRDQVTGSLKGRNDGDQPDSSEKNDRSPQPSSDESQ   50
ETASRTTQTRAGSRAARRRGKDKTTQTVEEPTPIETDEKPTNTKKQTRKK  100
EDRLVGRIVLIVVSVLVLMMAIFGFTFYKYVDAGLQPLDKNNKKLVQVHI  150
PEGSSNKQIAAVLEESNVIKSGMVFNYYVKFKNLTDFQAGYYQMSPSMTL  200
DEIGEMLKEGGTPEPTKIADGKVTIPEGYDIDKIGEAIEKNTDFKKADFI  250
ALMKNEDFFNQMKAKYPDLLESAATAEGVRYRLEGYLFPATYDYYKKATL  300
PEFVEQMIAKMNTVMEQYTPTIHAKNLTNQQVLTLASLVEKEGVKEADRK  350
QIAQVFFNRLAADMPIQSDISILYALGEHKETVTYADLEVDSSYNLYKNT  400
GYGPGPLDSPSEESIKAVLNPTPSDYLYFVADISTGKVYFSKTYEEHQVL  450
VDQYVNNSSSE                                         461
```

Figure S1. MltG protein sequence. MltG of *E. faecalis* (OG1RF_12215) is an integral membrane protein (transmembrane domain as predicted by TMHMM 2.0 is highlighted in blue) which contains two extracellular domains: the membrane proximal LysM, a putative peptidoglycan-binding domain highlighted in yellow, and the C-terminal YceG domain highlighted in green that catalyzes cleavage of nascent peptidoglycan. The key glutamate required for catalysis (E342) is highlighted in dark green. The N-terminal cytoplasmic domain contains 4 putative residues (highlighted in pink) for phosphorylation by IreK: T20, S49, T75, and T77. Site localization confidence for each residue was 94.55%, 81.87%, 34.6%, and 49.48%, respectively[7].

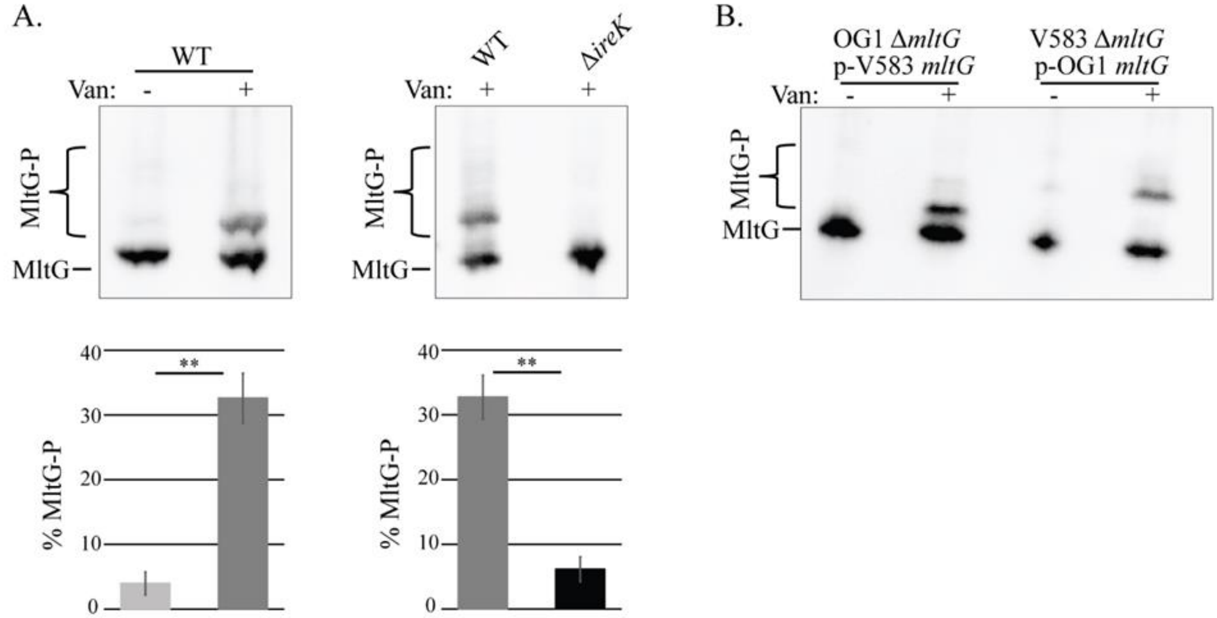

Figure S2. IreK-dependent phosphorylation of MltG in V583. Exponentially growing *E. faecalis* strains were exposed to 3 µg/mL vancomycin (van) for 20 min and whole cell lysates were prepared. Lysates were subjected to phos-tag SDS-PAGE with immunoblotting for MltG. The upper band(s) represent phosphorylated proteoform(s). (A) Signal intensities from the phosphorylated and unphosphorylated bands were used to calculate % phosphorylation of MltG, presented as the average % phosphorylation in bar graphs. n=3 and error bars represent +/- s.d. * = p<0.05; ** = p<0.01; ns = not significant. Student's t-test (heteroschidastic, two-tailed). (B) Expression of the MltG variants from V583 (p-V583 MltG) or OG1 (p-OG1 MltG) in the reciprocal Δ*mltG* host reveals that mobility of the phosphorylated MltG proteoform is determined by the identity of the MltG variant. Strains were WT = V583; Δ*ireK* = CK206.

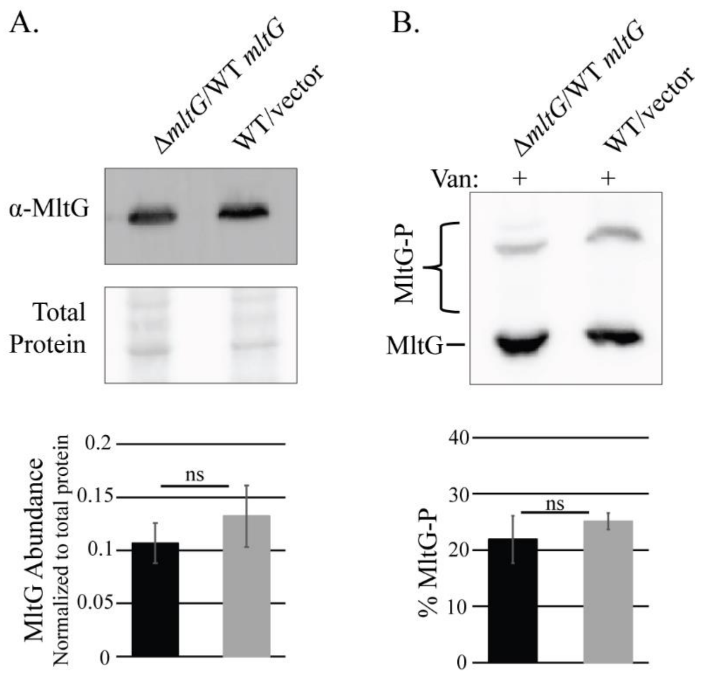

Figure S3: Plasmid-encoded MltG is expressed and phosphorylated normally.

(A) Ectopic expression of MltG from a plasmid was confirmed by immunoblot. Whole cell lysates of the designated strains grown to exponential phase in MHB were prepared. Lysates were subjected to SDS-PAGE supplemented with TCE for total protein detection and immunoblotting for MltG. Signal was normalized to total protein detected by TCE. (B) Phosphorylation of ectopically expressed MltG was compared to phosphorylation of chromosomally expressed MltG by phos-tag SDS-PAGE. Exponentially growing *E. faecalis* strains were exposed to 3 µg/mL vancomycin (van) for 20 min and whole cell lysates were prepared. Lysates were subjected to phos-tag SDS-PAGE with immunoblotting for MltG. The upper band(s) represent phosphorylated proteoform(s). Signal intensities from the phosphorylated and unphosphorylated bands were used to calculate % phosphorylation of MltG, presented as the average % phosphorylation in bar graphs. n=3 and error bars represent +/- s.d. * = p<0.05; ns = not significant. Student's t-test (heteroschidastic, two-tailed). Strains were WT/vector = OG1(pJRG9); Δ*mltG* /WT *mltG* = JL650(pAAU12).

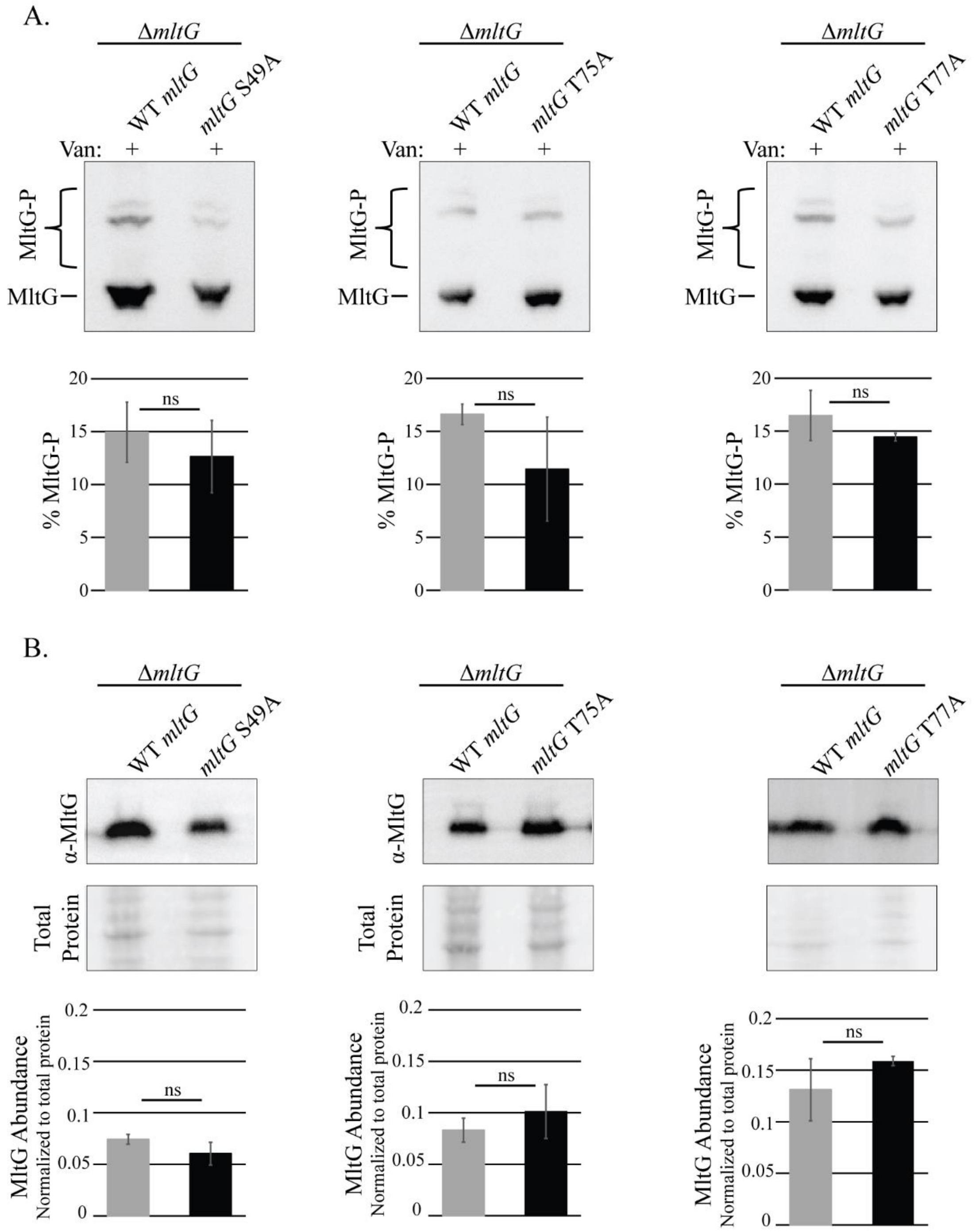

Figure S4: MltG S49A, T75A and T77A do not impact MltG phosphorylation. (A) Phosphorylation of ectopically expressed MltG variants were compared to WT by phos-tag SDS-PAGE. Exponentially growing *E. faecalis* strains were exposed to 3 μg/mL vancomycin (van) for 20 min and whole cell lysates were prepared. Lysates were subjected to phos-tag SDS-PAGE with immunoblotting for MltG. The upper

band(s) represent phosphorylated proteoform(s). Signal intensities from the phosphorylated and unphosphorylated bands were used to calculate % phosphorylation of MltG, presented as the average % phosphorylation in bar graphs compared to WT *mltG* expressed from the same plasmid. (B) Ectopic expression of MltG from plasmids was confirmed by subjecting whole cell lysates to SDS-PAGE supplemented with TCE for total protein detection and immunoblotting for MltG. Expression of all MltG mutants was compared to that of WT *mltG* expressed from the same plasmid. n=3 and error bars represent +/- s.d. * = $p<0.05$; ns = not significant. Student's t-test (heteroschidastic, two-tailed). Strains were Δ*mltG/*WT *mltG*= JL650(pAAU12); Δ*mltG*/*mltG* S49A = JL650(pAAK17); Δ*mltG/mltG* T75A = JL650(pAAK18); Δ*mltG/mltG* T77A = JL650(pAAK19).

Table S1. His$_6$-tagged MltG is functional

Strain[b]	Ceftriaxone MIC (µg/mL)[a]
WT/vector	128
Δ*mltG*/vector	512
Δ*mltG*/WT *mltG*	256
Δ*mltG*/WT *mltG-his$_6$*	128

[a]The median MIC of ceftriaxone determined from at least 3 biological replicates.

[b]Strains were WT/vector = OG1(pJRG9); Δ*mltG*/vector = JL650(pJRG9); Δ*mltG/*WT *mltG* = JL650(pAAU12); Δ*mltG/*WT *mltG*-his = JL650(pPS5).

Table S2. Strains and plasmids used in this study

Strain or plasmid	Description of genotype	Source or reference
**STRAINS**		
E. coli		
TOP10	Routine cloning host	Lab stock
DH5α	Routine cloning host	Lab stock
BL21(DE3)	Protein overexpression host	Lab stock
C43(DE3)	Protein overexpression host	Lucigen
E. faecalis		
OG1	Wild-type	Gold 1975[1]
JL650	OG1 Δ*mltG* (ΔQ6-D452)	Minton 2022[2]
JL206	OG1 Δ*ireK*	Labbe 2017[3]
JL455	OG1 Δ*ireP*	Labbe 2017[3]
V583	Wild-type, vancomycin-resistant clinical isolate	Sahm 1989[4]
CK206	V583 Δ*ireK*	Banla 2017[5]
**PLASMIDS**		
pET28a::his_6-smt3	his6-smt3 *E. coli* protein expression vector (Kn^r) with cleavable His6-SUMO tag	Brian Volkman Lab
pAAK42	pET28a::his_6-smt3-*mltG*	Knotek and Kristich, in press
pAAK56	pET28a::his_6-smt3-*mltG* 4A	This work
pAAK57	pET28a::his_6-smt3-*mltG* T20A	This work
pAAK58	pET28a::his_6-smt3-*mltG* T20E	This work
pAAK59	pET28a::his_6-smt3-*mltG* S49A T75A T77A	This work
pJRG9	*E. faecalis* expression vector, constitutive P23s promoter (Cm^r)	Snyder 2014[6]

pAAU12	pJRG9::WT *mltG*	Knotek and Kristich, in press
pAAK20	pJRG9::*mltG 4A*	This work
pAAK16	pJRG9::*mltG T20A*	This work
pAAK45	pJRG9::*mltG T20E*	This work
pAAK53	pJRG9::*mltG* S49A T75A T77A	This work
pAAK17	pJRG9::*mltG* S49A	This work
pAAK18	pJRG9::*mltG* T75A	This work
pAAK19	pJRG9::*mltG* T77A	This work
pPS5	pJRG9::*mltG*-his_6	This work

Supplemental References

1. Gold OG, Jordan HV, van Houte J. The prevalence of enterococci in the human mouth and their pathogenicity in animal models. *Arch Oral Biol*. 1975;20(7):473-IN15. doi:10.1016/0003-9969(75)90236-8

2. Minton N, Djoric D, Little J, Kristich CJ. GpsB promotes PASTA kinase signaling and cephalosporin resistance in Enterococcus faecalis. *J Bacteriol*. 2022;204(10). doi:https://doi.org/10.1128/jb.00304-22

3. Labbe BD, Kristich CJ. *Growth-and Stress-Induced PASTA Kinase Phosphorylation in Enterococcus Faecalis*.; 2017. https://doi.org/10

4. Sahm DF, Kissinger J, Gilmore MS, et al. In vitro susceptibility studies of vancomycin-resistant Enterococcus faecalis. *Antimicrob Agents Chemother*. 1989;33(9):1588-1591. doi:10.1128/AAC.33.9.1588

5. Banla IL, Kommineni S, Hayward M, et al. Modulators of Enterococcus faecalis cell envelope integrity and antimicrobial resistance influence stable colonization of the mammalian gastrointestinal tract. *Infect Immun*. 2018;86(1). doi:10.1128/IAI.00381-17

6. Snyder H, Kellogg SL, Skarda LM, Little JL, Kristich CJ. Nutritional control of antibiotic resistance via an interface between the phosphotransferase system and a two-component signaling system. *Antimicrob Agents Chemother*. 2014;58(2):957-965. doi:10.1128/AAC.01919-13

7. Iannetta AA, Minton NE, Uitenbroek AA, et al. IreK-Mediated, Cell Wall-Protective Phosphorylation in *Enterococcus faecalis*. *J Proteome Res*. Published online October 21, 2021:acs.jproteome.1c00635. doi:10.1021/acs.jproteome.1c00635
